# Comparative analysis of codon usage patterns in Rift Valley fever virus

**DOI:** 10.1590/1678-4685-GMB-2019-0240

**Published:** 2020-05-11

**Authors:** Hayeon Kim, Myeongji Cho, Hyeon S. Son

**Affiliations:** 1 Department of Biomedical Laboratory Science, Kyungdong University, Wonju, Gangwondo, Korea.; 2 Laboratory of Computational Biology & Bioinformatics, Institute of Public Health and Environment, Graduate School of Public Health, Seoul National University, Seoul, Korea.; 3 SNU Bioinformatics Institute, Interdisciplinary Graduate Program in Bioinformatics, College of Natural Science, Seoul National University, Seoul, Korea.

**Keywords:** Rift Valley fever virus, phylogenetic analysis, nucleotide composition, codon usage

## Abstract

Rift Valley fever virus (RVFV) is a vector-borne pathogen and is the most widely known virus in the genus *Phlebovirus*. Since it was first reported, RVFV has spread to western Africa, Egypt and Madagascar from its traditional endemic region, and infections continue to occur in new areas. In this study, we analyzed genomic patterns according to the infection properties of RVFV. Among the four segments of RVFV, the nucleotide composition, overall GC content and the difference of GC composition in the third position of the codons (%GC3) between groups were the largest in the S (NP) segment, showing that more diverse codons were used than in other segments. Furthermore, the results of CAI analysis of the S (NP) segment showed that viruses isolated from regions where no previous infections had been reported had the highest values, indicating greater adaptability to human hosts compared with other viruses. This result suggests that mutations in the S (NP) segment co-evolve with the infected hosts and may lead to expansion of the geographic range. The distinctive codon usage patterns observed in specific genomic regions of a group with similar infection properties may be related to the increasing likelihood of RVFV infections in new areas.

## Introduction

Recently, infection with Rift Valley fever virus (RVFV) was reported for the first time in China (Liu *et al.*, 2017). Although it was identified in a patient who was returning to China from Angola and was not directly infected in China, no RVFV infections have been reported in Angola previously (Liu *et al.*, 2017). Since its first report of infection and transmission between lambs in the Rift Valley of Kenya in 1930, RVFV continues to cause infections (Daubney *et al.*, 1931). Previously, RVFV infections were found mostly in parts of Africa such as Kenya, but infections are increasing outside the traditional endemic region, such as in the Middle East and Europe (Madani *et al.*, 2003; Chevalier *et al.*, 2010; Grobbelaar *et al.*, 2011). This trend indicates that RVFV is highly likely to cause infections in new areas.

RVFV is a vector-borne viral pathogen in the genus *Phlebovirus* and is known to cause zoonotic infections and change hosts via mosquitoes (*Aedes* spp., *Culex* spp., *Anopheles* spp., etc.) (Elliott, 1997; Bouloy and Weber, 2010). RVFV is an enveloped negative single-stranded RNA virus and ranges in size from 80 to 120 nm (Ellis *et al.*, 1979; Pepin *et al.*, 2010). RVFV has a circular three-segment genome, and these segments form a panhandle secondary structure due to cDNA sequences at the end of each segment (Hewlett *et al.*, 1977; Boshra *et al.*, 2011). Different proteins are encoded in each segment. The L segment encodes RNA polymerase used in the replication and mRNA transcription processes (Gerrard and Nichol, 2007). The M segment encodes two glycoproteins (Gn and Gc) that are required for viral entry and assembly and a nonstructural protein that inhibits cell apoptosis (Gerrard and Nichol, 2007). The S segment, with ambisense characteristics, encodes nucleoproteins that induce a host immune response in the antisense orientation, and nonstructural (NS) proteins that damage the host genome and function as an interferon antagonist in the complementary orientation (Gerrard and Nichol, 2007). Infections with RVFV can lead to serious illness, including retinitis, hepatitis, renal failure, meningoencephalitis, and severe hemorrhagic diseases, and can cause death in humans (Bird *et al.*, 2009). As there are currently no effective vaccines or treatments for RVFV, the emergence of RVFV in new areas may lead to serious public health problems (Faburay *et al.*, 2017). RVFV infection is mainly spread by mosquitoes, and therefore the area infected with RVFV is limited by the habitat distribution of its mosquito vectors (Tantely *et al.*, 2013). However, recent climate change and increasing international trade have resulted in migration and expanded habitat for the vectors, allowing RVFV infection to occur in unexpected areas (Chevalier *et al.*, 2010; Tantely *et al.*, 2013). In this study, we analyzed the infection properties of RVFV based on previously reported sequence information.

## Material and Methods

### Data collection

Sequence data was downloaded from the National Center for Biotechnology (NCBI) GenBank database (https://www.ncbi.nlm.nih.gov/genbank/) in order to compare the genetic characteristics of RVFVs that infect humans. RVFV sequences isolated from infected humans were studied in this analysis. Sequence datasets (in FASTA format) for the four coding-sequence (CDS) regions (large [L], medium [M], small [S] nonstructural protein [NS] and small [S] nucleoprotein [NP] segments) within each segment of the virus were grouped based on the region of collection (country) (Table 1).

**Table 1:** Summary of RVFV sequence characteristics.

Country	L segment	M segment	S (NP) segment	S (NS) segment
Kenya	10	2	14	14
Sudan	4	5	13	13
Central Africa Republic	2	7	8	7
Madagascar	6	6	6	6
Egypt	3	4	6	6
Angola	1	1	1	1
Saudi Arabia	1	2	2	1
China	3	3	3	3
Total	30	30	53	51

### Phylogenetic analysis

Phylogenetic analysis was performed on the L, M, and S (NP and NS) segments using the program MEGA7 (http://www.megasoftware.net) to examine the evolutionary relationships among RVFVs by region and time (year) (Kumar *et al.*, 2016). Sequence alignment was performed with MUSCLE in MEGA7, and the maximum likelihood (ML) method based on the Tamura-Nei model was used to construct phylogenetic trees (Tamura and Nei, 1993; Kumar *et al.*, 2016). A robustness test was conducted with the bootstrap value set to 1,000.

### Codon usage analysis

Analysis of codon usage bias in viruses provides information on molecular evolution; it can also improve understanding of the regulation of viral gene expression and help to identify the efficient expression process of viral proteins required to evade immune responses (Shackelton and Holmes, 2004; Butt *et al.*, 2014). In this study, genomic patterns were compared by analyzing the nucleotide composition features of each segment, and codon usage bias was evaluated using the effective number of codons (ENC). The ENC value is 20 if only one synonymous codon is preferred and ranges up to 61 if all synonymous codons are equally preferred (Wright, 1990). There is an inverse relationship between ENC and gene expression. A lower ENC value indicates strong codon usage bias and elevated gene expression, while a higher ENC value indicates a diversity of codons encoding amino acids and lower gene expression (Wright, 1990). Generally, an ENC value > 35 suggests that there is a relatively conserved genomic composition (Comeron and Aguadé, 1998). Furthermore, differences in the preference of codons for a single amino acid were examined using relative synonymous codon usage (RSCU) values (Sharp and Li, 1986). Amino acids can be simultaneously encoded by one to six different codons, and codons encoding the same amino acid tend show preferential usage (Plotkin *et al.*, 2006). Generally, codons with RSCU values > 1.0 are more preferred (abundant codons), while those with RSCU values < 1.0 are less preferred (less-abundant codons). An RSCU value of 1.0 indicates that all codons were used randomly or equally (Sharp and Li, 1986). In this study, codon usage patterns were analyzed using the tools of the Gene Infinity website (http://www.geneinfinity.org/sms/sms_codonusage.html), and codon adaptation index (CAI) values were calculated for comparison of general codon usage patterns among the virus and its hosts, human and mosquito, using the CAIcal program (ver. 1.4, http://genomes.urv.cat/CAIcal).

## Results

### Phylogenetic relationships and classification of RVFV

**Figure 1:**
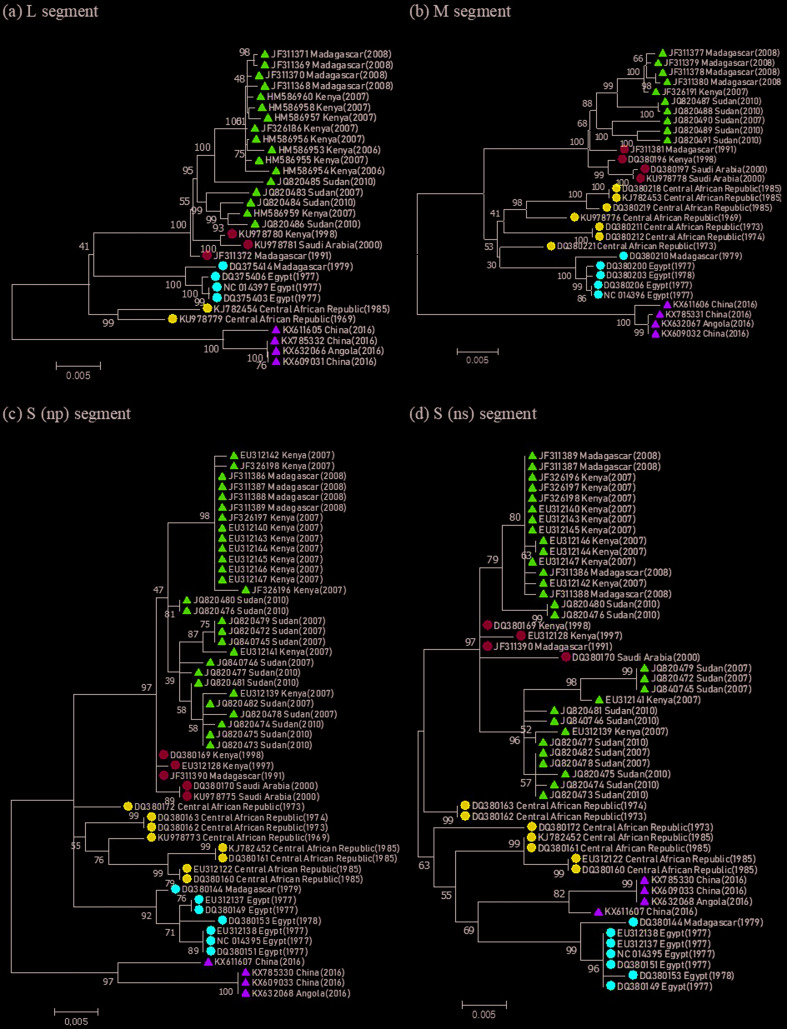
Phylogenetic analysis of RVFV; (a) L segment, (b) M segment, (c) S (np) segment, (d) S (ns) segment RVFVs were classified into five groups (Group 1: [pink triangle]; Group 2: [sky blue circle]; Group 3: [dark blue circle]; Group 4: [red circle]; Group 5: [light green triangle]).

Phylogenetic trees were constructed for each segment (S [NS, NP], M, and L) of the RVFV genome. RVFVs were grouped according to the infected region (country) and time (year) in the constructed trees (Figure 1). This result indicated that RVFVs do not cause infections with the same genetic composition, but rather the genomic features of this virus vary with region and time due to mutations, which can also lead to changes in viral infection patterns. RVFV infections do not maintain the same level of toxicity every year, and the reported death rate due to the virus varies according to the time and region. In particular, the RVFV sequences from Kenya and Sudan in 2007–2008 that were subjected to analysis were found to form a single group, and RVFV infection caused a considerable number of deaths in Kenya (155 deaths; case fatality rate 23%) and Sudan (230 deaths; case fatality rate 30.8%) (World Health Organization, 2007; Hassan *et al.*, 2011). This result shows that mutations in RVFV may affect its toxicity. Although, the genetic lineages (A~G) of RVFV have been classified by previous studies (Bird *et al.*, 2007; Ikegami, 2012), the groups of RVFV in this study were re-classified based on the phylogenetic analysis for the collected sequences. This is because previous studies did not consider the sequences of RVFV that occurred in the 2000s. Therefore, we based on these results, codon usage patterns of the five groups of RVFV (Group 1: Kenya [2006–2007], Madagascar [2008], and Sudan [2010]; Group 2: Madagascar [1991], Kenya [1998], and Saudi Arabia [2000]; Group 3: Central African Republic [1969 and 1985]; Group 4: Egypt [1997] and Madagascar [1979]; and Group 5: Angola [2016] and China [2016]) were analyzed and compared.

### Nucleotide composition of the CDS region in RVFV

Four CDS regions were analyzed for each segment to compare the nucleotide compositions of the five groups identified in phylogenetic analysis (Table 2). In the L, M and S (NS) segments, no significant difference in base composition was detected. In contrast, the nucleotide composition features of each group in the S (NP) segment showed a difference in composition of the third base. The third bases A (A3), C (C3), T (T3), and G (G3) of the S (NP) segment had overall frequencies in the range of 17.48–21.09%, 21.22–23.48%, 25.61–27.66%, and 29.73–33.44%, respectively. These results show that among the four CDS regions of RVFV, the S (NP) segment may be a useful indicator for identifying the genetic properties of RVFVs.

**Table 2:** Nucleotide composition of RVFV segments.

		A1	A2	A3	C1	C2	C3	T1	T2	T3	G1	G2	G3
L	Group 1	31.67	32.13	25.08	16.72	20.78	21.56	21.56	30.27	28.88	30.04	16.82	24.48
	Group 2	31.61	32.08	25.34	16.67	20.83	21.66	21.60	30.24	28.68	30.12	16.85	24.32
	Group 3	31.39	31.88	24.95	16.80	20.88	21.85	21.48	30.24	28.48	30.34	17.00	24.72
	Group 4	31.56	32.01	25.49	16.75	20.83	21.62	21.50	30.24	28.69	30.20	16.91	24.20
	Group 5	31.63	32.01	24.63	16.65	20.89	21.38	21.53	30.18	29.12	30.20	16.92	24.87
M	Group 1	28.36	28.14	24.34	17.19	24.04	21.09	22.46	27.46	30.30	31.99	20.36	24.27
	Group 2	28.51	28.05	23.89	17.15	23.96	20.49	22.48	27.61	30.86	31.87	20.39	24.75
	Group 3	28.39	28.11	24.30	17.27	23.93	21.00	22.36	27.58	30.18	31.98	20.38	24.51
	Group 4	28.35	28.11	24.22	16.96	23.96	20.93	22.77	27.58	30.32	31.92	20.35	24.53
	Group 5	28.40	28.19	24.46	17.26	24.00	20.70	22.39	27.52	31.07	31.95	20.28	23.77
S (NP)	Group 1	28.05	31.30	19.07	21.17	26.02	21.22	15.01	27.24	27.66	35.77	15.45	32.06
	Group 2	28.05	31.30	19.11	20.73	26.02	21.62	15.45	27.24	27.16	35.77	15.45	32.11
	Group 3	28.05	31.20	21.09	20.83	26.02	21.80	15.35	27.24	27.39	35.77	15.55	29.73
	Group 4	28.52	30.89	20.50	20.15	26.02	22.24	15.62	27.24	26.95	35.71	15.85	30.31
	Group 5	28.05	31.30	17.48	21.04	26.02	23.48	15.14	27.24	25.61	35.77	15.45	33.44
S (NS)	Group 1	25.18	26.15	20.72	23.53	20.65	21.74	17.40	34.60	35.78	33.90	18.60	21.76
	Group 2	25.19	26.32	20.68	23.59	20.68	21.90	17.39	34.59	35.53	33.83	18.42	21.90
	Group 3	25.08	26.32	20.41	23.52	20.68	21.70	17.45	34.59	35.82	33.94	18.42	22.07
	Group 4	25.19	26.27	20.30	23.68	20.68	21.38	17.67	34.59	35.76	33.46	18.47	22.56
	Group 5	25.10	26.32	19.27	23.68	20.59	21.43	17.39	34.68	36.75	33.83	18.42	22.56

### Compositional properties of the CDS region of RVFV

The %GC, %GC3, ENC, and CAI values were calculated for each group in order to analyze codon usage patterns in RVFVs. The %GC and %GC3 values showed the most significant differences between groups within the S (NP) segment (Table 3). The %GC3 value indicates the frequency of occurrence of guanine (G) or cytosine (C) at the wobble site, which is the third position of a codon. The %GC3 values were found to be greater than 50% for all groups in the S (NP) segment, but less than 50% in the other three segments. This result shows that the frequency of codons ending in G or C is higher than that of adenine (A) or thymine (T). In particular, the %GC3 values of the five groups were 51.50–56.90%, showing a greater difference between groups than other segments. The CDS region of the S (NP) segment encodes a nucleoprotein, and nucleoprotein of RVFV is known to induce host immune responses. This finding suggests that differences in host immune responses to the virus and the varied outcome of viral infection for each group may be caused by the properties of the S (NP) segment. As a result of ENC analysis, RVFV was found to have a high ENC value overall. Although the difference between groups was not great, the ENC value of the S (NP) segment was notably high (> 60), indicating that the CDS region of the S (NP) segment uses a greater variety of codons than other CDS regions. The CAI value is a measure of similarity in the codon usage pattern of a given gene, with that of the host species used as a reference. This study used the CAI values of the mosquito (*Aedes aegypti*), a representative vector of RVFV, and the infected host (*Homo sapiens*) for comparison of general codon usage patterns. As the CAI value approaches one, the codon usage pattern becomes more similar to that of the reference individual. Overall, the CAI value with humans (*Homo sapiens*) as a reference was higher than that with mosquitos (*Aedes aegypti*). Remarkably, the CAI value of the S (NP) segment is highest in Group 5. In this study, all viral data for Group 5 were obtained from RVFVs collected in 2016. These viruses were isolated from new regions (Angola and China) where no previous cases of infection were reported, and the viral data used for analysis is the most recent data among the five groups. These results suggest that mutations in the S (NP) segment co-evolve with the hosts (mosquitoes and humans) and may allow the virus to expand its geographic range.

**Table 3:** %GC, %GC3, ENA and CAI values of RVFV segments.

		%GC	%GC3	ENC	CAI^a^	CAI^b^
L segment	Group 1	43.46	46.03	51.29	0.764	0.693
	Group 2	43.50	46.00	51.43	0.763	0.691
	Group 3	43.88	46.55	51.45	0.764	0.697
	Group 4	43.50	45.85	51.30	0.763	0.690
	Group 5	43.63	46.25	51.30	0.765	0.688
M segment	Group 1	46.32	45.34	49.49	0.762	0.666
	Group 2	46.20	45.25	49.58	0.764	0.666
	Group 3	46.34	45.49	49.74	0.761	0.665
	Group 4	46.22	45.46	50.00	0.765	0.668
	Group 5	45.98	44.48	49.98	0.761	0.671
S (NP) segment	Group 1	50.55	53.27	60.32	0.748	0.696
	Group 2	50.54	53.78	60.82	0.753	0.696
	Group 3	49.90	51.50	60.51	0.748	0.688
	Group 4	50.09	52.53	60.99	0.747	0.700
	Group 5	51.75	56.90	61.00	0.762	0.716
S (NS) segment	Group 1	46.73	43.53	55.29	0.745	0.693
	Group 2	46.75	43.80	55.25	0.745	0.695
	Group 3	46.79	43.77	54.54	0.754	0.703
	Group 4	46.70	43.94	55.64	0.743	0.698
	Group 5	46.85	44.00	53.23	0.754	0.704

**Figure 2:**
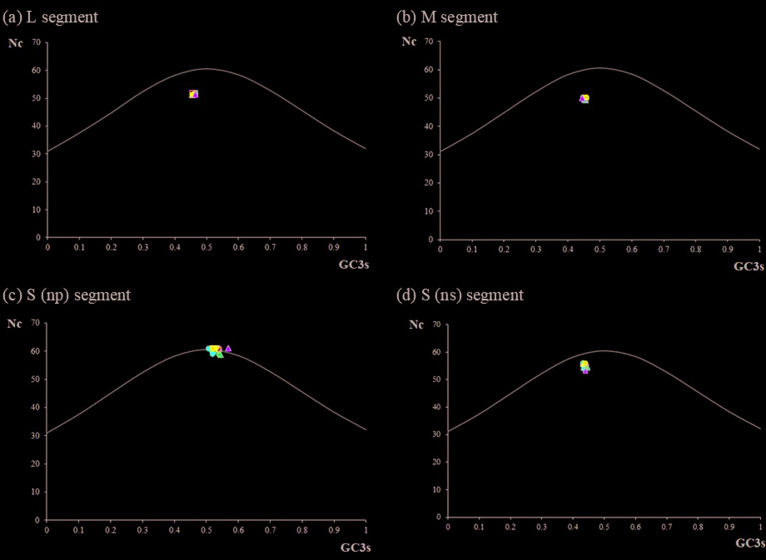
ENC versus GC3 plots for RVFV segments; (a) L segment, (b) M segment, (c) S (np) segment, (d) S (ns) segment ENC plotted against GC3 of the CDS regions in the L, M and S (NS) segments are distributed below the curve, which means that codon usage is biased. In the S (NP) segment, the ENC values are distributed above the curve, showing that codon usage is more variable.

In addition, ENC plots were generated for each CDS region in order to determine the degree of compositional constraints on codon usage bias in the RVFVs (Figure 2). The ENC plot shows variation of ENC values according to the change in %GC3 as a decentralized graph and is known to be an effective method for examining codon usage variations among genes. In the present study, ENC values plotted against %GC3 of the CDS regions in the L, M and S (NS) segments were distributed below the curve, showing that codon usage is biased. In contrast, for the S (NP) segment, the ENC values were distributed above the curve, indicating that codon usage is more variable.

### Prevalence of preferred codons

**Figure 3:**
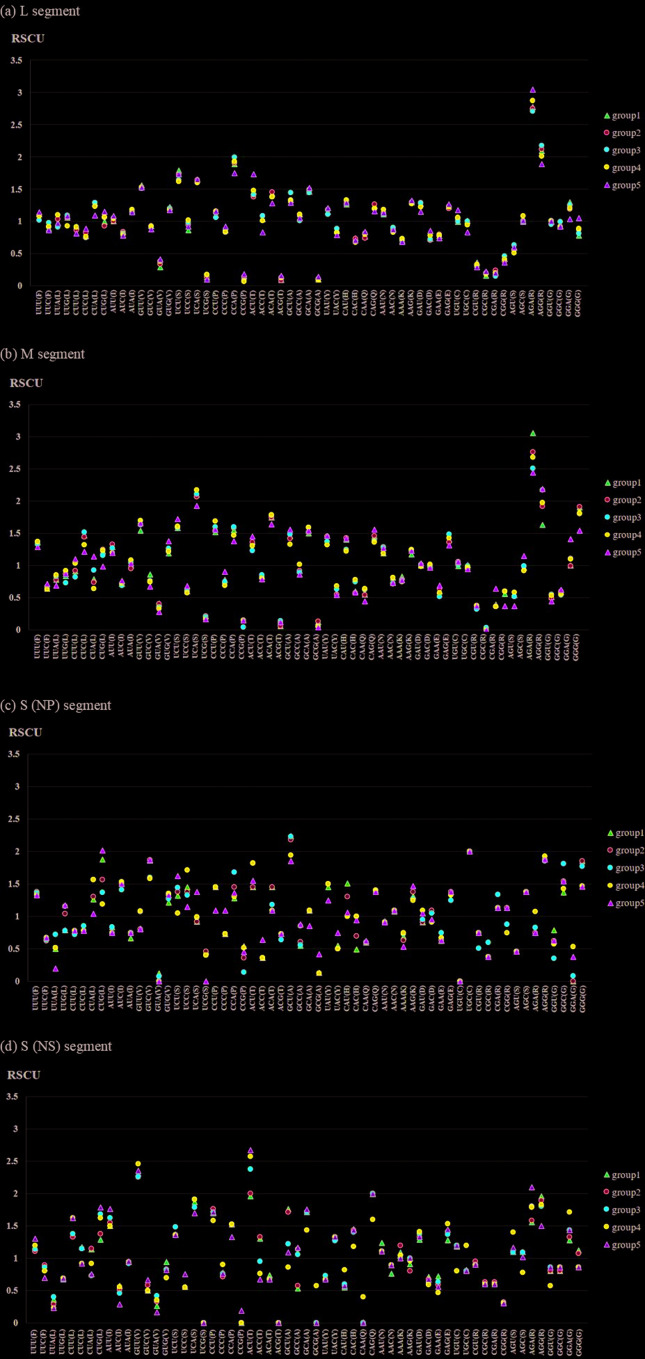
RSCU analysis of RVFV segments; (a) L segment, (b) M segment, (c) S (np) segment, (d) S (ns) segment There is more variation in the differences of codon preferences between groups in the S segment compared to the L and M segments.

RSCU analysis was performed to determine whether group-specific properties could be discriminated from differing codon preferences in each CDS region (Figure 3). In the L segment, the codons AGC (R) and AGG (R) showed relatively large differences in RSCU values compared to other codons and were found to be over-represented. Most other codons showed similar preferences, with the same over-represented codons (≥ 1.6) and under-represented codons (≤ 0.6) and no differences among groups. In the M segment, the codons AGC (R), AGG (R) and UCA (S) showed relatively large differences in RSCU values compared to other codons and were identified as over-represented codons. The RSCU values of the codons CGA (R), CGG (R) and GGG (G) in Groups 1 to 4 were 0.36–0.39, 0.56–0.6 and 1.8–1.91, while those in Group 5 were 0.64, 0.37, and 1.54, respectively, indicating large differences compared to other groups. In the S (NP) segment, UUA (L) was an under-represented codon except in Group 3 (0.72) and had the lowest representation in Group 5 (0.2), while CUG (L) was identified as an over-represented codon in Group 1 (1.88) and Group 5 (2.02). In Group 5, the most highly preferred codon was UCU (S), with RSCU values ≥ 1.6 (1.62), while the RSCU value of the codon UCG (S) was 0.0, showing a different codon usage pattern from other groups. In Group 1, the RSCU values of the codons CAU (H) and CAC (H) were 1.51 and 0.49, respectively, indicating differences in codon preference from other groups. In Group 3, the RSCU values of the codons GGU (G) and GGC (G) were 0.35 and 1.81, respectively, showing a different codon usage pattern from other groups. In the S (NS) segment, CUU (L) was an over-represented codon in Group 4 (1.62) and Group 5 (1.62), and GUG (L) showed a significant difference in codon preference in Group 3 (1.68), Group 4 (1.62), and Group 5 (1.79). The RSCU values of the codon GCU (A) were 1.71 (Group 1) and 1.76 (Group 2), while those of codon GCC (A) were 0.53 (Group 1) and 0.57 (Group 2), respectively. In Group 4, the RSCU values of GCA (A) and GCC (A) were 1.43 and 0.57, respectively, indicating a difference in codon preferences compared to other groups. RSCU analysis showed that the difference in codon preference between groups was more variable in the S segment than in the L and M segments.

## Discussion

Various factors allow viruses to expand their range and rapidly evolve pathogenicity when adapting to new environments and hosts, including natural environmental factors and anthropogenic factors, such as climate change and the development of international trade and transportation. Surveillance of the emergence of viruses is important, as an unexpected influx of new infectious agents into a new area can cause serious illnesses in unimmunized populations. RVFV infections are being reported in new areas continuously and constant monitoring for the emergence of the virus is required. This study investigated whether the effects of RVFV on hosts differed among epidemic periods and whether evolutionary changes in viruses are involved in the expansion of the affected area. RVFVs were grouped based on the collection time and region through phylogenetic analysis. Based on the sample clusters, nucleotide composition and codon usage were analyzed. The nucleotide composition, overall GC content, and differences in GC content in the third codon position (%GC3) between groups were greatest in the S (NP) segment, confirming that more diverse codons were used there than in other segments. Remarkably, in CAI analysis of the S (NP) segment, Group 5 had the highest value, indicating that Group 5 viruses have the greatest similarity to the reference data in terms of codon usage patterns and expression levels, and that they are better adapted to human hosts compared with other groups. Group 5 consisted of the most recent viral samples among the five groups, and all Group 5 viruses were isolated from new regions (Angola and China) where no previous cases of infection had been reported. These results suggest that mutations in the S (NP) segment co-evolve with infected hosts, i.e., mosquitoes and humans, and may lead to expansion of the areas where viral infection occurs. Due to the limitations of the published data, we could not analyze some recently isolated RVFV sequence data, other than data for Group 5 collected in Angola and China. Sufficient genetic data can reduce the bias that can occur during the analysis, so if a future analysis is performed using additional public data, it may provide important information to confirm the relationship between evolutionary variation in the patterns of RVFV and the incidence of infection. RVFV viruses have a relatively large number of conserved genomic regions, and infection has occurred mainly in limited areas due to the geographically limited habitat of its vectors. However, the results of this study showed distinct codon usage patterns in specific genomic regions and identified a group of RVFVs that might have an increased possibility of causing infections in new areas based on genetic mutations. Therefore, continuous monitoring of RVFV is necessary to prevent an epidemic of this infectious disease. The codon usage patterns of RVFVs demonstrated in the present study suggest the need for continuous monitoring of RVFV infections, particularly with regard to mechanisms of viral evolution and adaptation to new environmental conditions and to human hosts.

## References

[B1] Bird BH, Khristova ML, Rollin PE, Ksiazek TG, Nichol ST (2007). Complete genome analysis of 33 ecologically and biologically
diverse Rift Valley fever virus strains reveals widespread virus movement
and low genetic diversity due to recent common ancestry. J Virol.

[B2] Bird BH, Ksiazek TG, Nichol ST, Maclachlan NJ (2009). Rift Valley fever virus. J Am Vet Med Assoc.

[B3] Boshra H, Lorenzo G, Busquets N, Brun A (2011). Rift valley fever: recent insights into pathogenesis and
prevention. J Virol.

[B4] Bouloy M, Weber F (2010). Molecular biology of rift valley Fever virus. Open Virol J.

[B5] Butt AM, Nasrullah I, Tong Y (2014). Genome-wide analysis of codon usage and influencing factors in
Chikungunya viruses. PLoS One.

[B6] Chevalier V, Pépin M, Plée L, Lancelot R (2010). Rift Valley fever—a threat for Europe?. Euro Surveill.

[B7] Comeron JM, Aguadé M (1998). An evaluation of measures of synonymous codon usage
bias. J Mol Evol.

[B8] Daubney R, Hudson JR, Garnham PC (1931). Enzootic hepatitis or rift valley fever. An undescribed virus disease of sheep cattle and man from East Africa. J
Pathol.

[B9] Elliott RM (1997). Emerging viruses: the Bunyaviridae. Mol Med.

[B10] Ellis DS, Simpson DI, Stamford S, Abdel Wahab KS (1979). Rift Valley fever virus: some ultrastructural observations on
material from the outbreak in Egypt 1977. J Gen Virol.

[B11] Faburay B, LaBeaud AD, McVey DS, Wilson WC, Richt JA (2017). Current status of rift valley fever vaccine
development. Vaccines.

[B12] Gerrard SR, Nichol ST (2007). Synthesis, proteolytic processing and complex formation of
N-terminally nested precursor proteins of the Rift Valley fever virus
glycoproteins. Virology.

[B13] Grobbelaar AA, Weyer J, Leman PA, Kemp A, Paweska JT, Swanepoel R (2011). Molecular epidemiology of Rift Valley fever virus. Emerg Infect Dis.

[B14] Hassan OA, Ahlm C, Sang R, Evander M (2011). The 2007 Rift Valley fever outbreak in Sudan. PLoS Negl Trop Dis.

[B15] Hewlett MJ, Pettersson RF, Baltimore D (1977). Circular forms of Uukuniemi virion RNA: an electron microscopic
study. J Virol.

[B16] Ikegami T (2012). Molecular biology and genetic diversity of Rift Valley fever
virus. Antiviral Res.

[B17] Kumar S, Stecher G, Tamura K (2016). MEGA7: Molecular Evolutionary Genetics Analysis Version 7.0 for
bigger datasets. Mol Biol Evol.

[B18] Liu J, Sun Y, Shi W, Tan S, Pan Y, Cui S, Zhang Q, Dou X, Lv Y, Li X (2017). The first imported case of Rift Valley fever in China reveals a
genetic reassortment of different viral lineages. Emerg Microbes Infect.

[B19] Madani TA, Al-Mazrou YY, Al-Jeffri MH, Mishkhas AA, Al-Rabeah AM, Turkistani AM, Al-Sayed MO, Abodahish AA, Khan AS, Ksiazek TG (2003). Rift Valley fever epidemic in Saudi Arabia: epidemiological,
clinical, and laboratory characteristics. Clin Infect Dis.

[B20] Pepin M, Bouloy M, Bird BH, Kemp A, Paweska J (2010). Rift Valley fever virus (Bunyaviridae: Phlebovirus): an update on
pathogenesis, molecular epidemiology, vectors, diagnostics and
prevention. Vet Res.

[B21] Plotkin JB, Dushoff J, Desai MM, Fraser HB (2006). Codon usage and selection on proteins. J Mol Evol.

[B22] Shackelton LA, Holmes EC (2004). The evolution of large DNA viruses: combining genomic information
of viruses and their hosts. Trends Microbiol.

[B23] Sharp PM, Li WH (1986). Codon usage in regulatory genes in Escherichia coli does not
reflect selection for ‘rare’ codons. Nucleic Acids Res.

[B24] Tamura K, Nei M (1993). Estimation of the number of nucleotide substitutions in the
control region of mitochondrial DNA in humans and
chimpanzees. Mol Biol Evol.

[B25] Tantely ML, Rakotoniaina JC, Tata E, Andrianaivolambo L, Razafindrasata F, Fontenille D, Elissa N (2013). Biology of mosquitoes that are potential vectors of Rift Valley
Fever virus in different biotopes of the central highlands of
Madagascar. J Med Entomol.

[B26] World Health Organization (2007). Outbreaks of Rift Valley fever in Kenya, Somalia, and United
Republic of Tanzania, December 2006-April 2007. Wkly Epidemiol Rec.

[B27] Wright F (1990). The ‘effective number of codons’ used in a gene. Gene.

